# Toxic Effects of Domoic Acid in the Seabream *Sparus aurata*

**DOI:** 10.3390/md8102721

**Published:** 2010-10-15

**Authors:** Isabel Nogueira, Alexandre Lobo-da-Cunha, António Afonso, Socorro Rivera, Joana Azevedo, Rogério Monteiro, Rosa Cervantes, Ana Gago-Martinez, Vítor Vasconcelos

**Affiliations:** 1 CIIMAR/CIMAR, Centro Interdisciplinar de Investigação Marinha e Ambiental, Rua dos Bragas 177-289, 4150-123 Porto, Portugal; E-Mails: isabelcaldevilla@gmail.com (I.N.); aafonso@icbas.up.pt (A.A.); joana_passo@hotmail.com (J.A.); 2 Laboratório de Biologia Celular, Instituto de Ciências Biomédicas Abel Salazar, ICBAS, Lg. Abel Salazar 2, 4099-003 Porto, Portugal; E-Mail: alcunha@icbas.up.pt (A.L.C.); 3 Departamento de Química Analítica, Facultad de Química, As Lagoas Marcosende C.P. 36210, Spain; E-Mails: srivera@uvigo.es (S.R.); rosynac@uvigo.es (A.G.-M.); 4 Laboratório de Histologia e Embriologia, Instituto de Ciências Biomédicas Abel Salazar, ICBAS, Lg. Abel Salazar 2, 4099-003 Porto, Portugal; E-Mail: rafmont@icbas.up.pt (R.M.); 5 Departamento de Biologia, Faculdade de Ciências, Universidade do Porto, Rua do Campo Alegre 4169-007 Porto, Portugal

**Keywords:** domoic acid, amnesic shellfish poisoning, Pseudo-nitzschia, algal neurotoxin, Sparus aurata

## Abstract

Neurotoxicity induced in fish by domoic acid (DA) was assessed with respect to occurrence of neurotoxic signs, lethality, and histopathology by light microscopy. *Sparus aurata* were exposed to a single dose of DA by intraperitoneal (i.p.) injection of 0, 0.45, 0.9, and 9.0 mg DA kg^−1^ bw. Mortality (66.67 ± 16.67%) was only observed in dose of 9.0 mg kg^−1^ bw. Signs of neurological toxicity were detected for the doses of 0.9 and 9.0 mg DA kg^−1^ bw. Furthermore, the mean concentrations (±SD) of DA detected by HPLC-UV in extracts of brain after exposure to 9.0 mg DA kg^−1^ bw were 0.61 ± 0.01, 0.96 ± 0.00, and 0.36 ± 0.01 mg DA kg^−1^ tissue at 1, 2, and 4 hours. The lack of major permanent brain damage in *S. aurata,* and reversibility of neurotoxic signs, suggest that lower susceptibility to DA or neuronal recovery occurs in affected individuals.

## 1. Introduction

Domoic acid (DA) is synthesized by marine algae, such as the red algae *Chondria armata* and species of the diatom genus *Pseudo-nitzschia* [1,2]. DA is an analogue of kainic acid (KA), which shows excitotoxic activity. Both DA and KA are structurally similar to glutamate, which is the predominant neurotransmitter in the central nervous system. This structural similarity allows DA and KA to bind to the glutamate receptor [GluRs] family, inducing neuroexcitatory and neurotoxic effects [3–5].

The first documented case of DA intoxication occurred in 1987, when at least four people died and over a hundred others suffered severe neurological and gastrointestinal disorders [6]. Since then, other DA intoxication events have been recorded in the marine community, resulting in mass mortalities of sea birds and sea lions [7–10]. DA has been recognized as a harmful food web-transferred phycotoxin to humans, through the consumption of mussels [11] and to marine mammals and birds, through planktivorous fish and krill [12,13]. Although neurotoxic effects that result from the consumption of DA have been described in mammals and birds, to date no field evidence of DA toxicity in fish has been documented. Nevertheless, concentrations of 39 ± 0.7 mg DA kg^−1^ in the body tissues of anchovies (*Engraulis mordax*) have been found [8].

Thus far, DA-induced neurotoxicity in fish is poorly understood and the majority of the available information is related to DA-induced excitotoxicological signs that appeared following the intraperitoneal (i.p.) injection of DA. When juvenile leopard sharks (*Triakis semifasciata*) were injected intraperitoneally with doses that ranged from 9–27 mg DA kg^−1^ body weight (bw), no signs of toxicity were observed [14]. In contrast, the i.p. injection of doses from 1–14 mg DA kg^−1^ bw induced excitotoxicological signs and death in anchovies (*E. mordax*) [15]. Further evidence of DA neurotoxicity in fish was also obtained after the i.p. injection of killifish (*Fundulus heteroclitus*) with 5 mg DA kg^−1^ bw, which led to behavioral alterations and increased the neuronal expression of c-Fos [16]. Similarly, the i.p. injection of coho salmon (*Oncorhynchus kisutch*) with doses of 6.3 ± 0.6 mg DA kg^−1^ bw resulted in neurotoxic signs [17]. In addition, changes in metabolic activity in the brain of the Atlantic salmon (*Salmo salar*) were found after the i.p. injection of 6.0 mg DA kg^−1^ bw [8].

To summarize, in the literature there is evidence that DA neurotoxicity susceptibility in fish, following intraperitoneal (i.p.) injection, varies within species. Therefore, the main objective of this study was to evaluate DA-induced neurotoxicity in another model, the gilthead seabream *Sparus aurata*. This species is common in the Mediterranean Sea, present along the Eastern Atlantic coasts from Great Britain to Senegal, both in marine and brackish waters such as coastal lagoons and estuarine areas, and feed on a variety of prey, such as shellfish, that can concentrate DA during *Pseudo-nitz*schia blooms. For this purpose, DA-induced neurotoxicity was assessed by analyzing mortality, the occurrence of neurotoxic signs and histopathological changes. The distribution of glutamate receptors 5, 6, and 7 (GluR5, 6, 7) in the brain of *S. aurata* was also studied by microscopy. In addition, the relationship between the neurotoxic signs was observed and the areas of the brain in *S. aurata* that were affected by DA were analyzed. The amount of DA in the liver and brain of *S. aurata* after exposure to DA was also evaluated.

## 2. Materials and Methods

### 2.1. Chemicals

All solvents and chemical reagents were high performance liquid chromatography (HPLC) or analytical grade. Methanol (Merck) and acetonitrile (Pancreac) were HPLC grade; perchloric acid (Pancreac) and formic acid (Prolabo) were analytical grade. DA (95% purity) was purchased from Sigma-Aldrich. Milli-Q water (Millipore Corporation) was used to prepare all solutions of the toxin.

### 2.2. Fish maintenance

Juvenile seabream (*S. aurata*) was used as a model species because of its ability to yield reproducible behavior data under controlled conditions [19].

*S. aurata* were supplied by a commercial fish farm (TIMAR Lda., Setubal, Portugal), where they were raised until they reached 10–15 g. Fish were kept at our laboratory for two months, during which time they were fed three times a week with a maintenance ration of 2–3% bw, before being used in experiments.

*S. aurata* were acclimatized to the experimental conditions for 48 h in 150-L glass aquaria. Food was not provided during the acclimation phase or during the course of the experiment. Aeration was provided through plastic tips placed 2 cm above the bottom of the aquarium.

### 2.3. Experimental design

The experiments were done in natural seawater (34 ± 0.7 ppm) under a photoperiod of 12 h light:12 h dark at 17 ± 0.8 °C. The amount of dissolved oxygen (68 ± 22%), pH (7.7 ± 0.1), and concentration of total ammonia (0.2 ± 0.1 mg L^−1^) were monitored daily. All aquaria that contained test organisms were isolated with opaque plastic to reduce external stimuli, such as movement of the experimenter, vibration, or visual cues.

A maximum of six fish per 36 L aquarium were used in the experiments, and, in order to account for the intergroup aquarium variability, there were always three aquaria per treatment and two controls. Controls were individuals not injected or injected with phosphate buffered saline (PBS). These were used in order to evaluate non-toxic changes in the fish caused by manipulation, induced stress, or the effects of PBS.

DA was dissolved in PBS and the stock solution was kept at −20 °C until the experimental solutions were prepared.

All fish were anesthetized within two minutes by immersion in 2-phenoxyethanol (0.2 mL L^−1^), before being weighed, then the i.p. injection volume was calculated in order to obtain the desired concentration.

At the end of each experiment, the water from each aquarium was renewed. The experimental setup used to evaluate the effects of DA in the gilthead seabream is summarized in Table 1.

The fish were handled in accordance with the guidelines for accommodation and care of animals from the European Convention for the Protection of Vertebrate Animals used for Experimental and Other Scientific Purposes.

### 2.4. Examination of DA neurotoxicity

Eighteen individuals were exposed to 0.0, 0.45, 0.9, or 9.0 mg DA kg^−1^ bw or PBS by i.p. injection. We conducted different consecutive studies within a period of one month, testing different treatments at the same time, using the nominal above mentioned concentrations.

DA-induced neurotoxicity was assessed by mortality and the occurrence of neurotoxic signs, such as swimming in a circle, in a spiral, or upside down. Digital videos were recorded for 20 minutes, starting 30 minutes and two hours after the exposure. Moreover, mortality was evaluated after 24 hours of exposure. Given that ≥50% mortality was detected in the fish injected with 9.0 mg DA kg^−1^ bw, no higher doses of DA were used in the study.

### 2.5. Light microscopy and immunohistochemistry

Six individuals exposed to 0.0, 0.45, 0.9, or 9.0 mg DA kg^−1^ bw or PBS were killed by an overdose of anesthetic after 24 hours of exposure. The brain, liver, stomach and duodenum were collected and fixed in formaldehyde (4%). These tissues were processed routinely for embedding in paraffin, sectioned at 4 μm, and stained with hematoxylin and eosin. Sections of the metencephalon (more specifically, the cerebellar cortex), diencephalon, myelencephalon, and mesencephalon from individuals exposed to PBS for 24 hours were mounted on slides, treated with 3-aminopropyltriethoxysilane (Sigma) to improve adhesion of the sections, and used for immunohistochemical studies using a previously described method [14].

A streptavidin-biotin-peroxidase immunohistochemistry kit (Histostain Plus; Zymed) was used to detect binding of the primary antibody, according to the manufacturer’s instructions with minor adaptations. After rinsing in distilled water and PBS, the peroxidase activity was visualized using 0.05% 3,3-diaminobenzidine (DAB) in PBS and 0.03% H_2_O_2_, which gave a brown-colored product. After rinsing in tap water, the sections were mounted in DPX. The primary monoclonal antibody MAB379, which recognizes an epitope common to GluR5, 6, and 7, was purchased from Chemicon International (Temecula). Negative controls were performed in which no primary antibody was added; mouse brain tissue was used as a positive control. Positive immunoreactivity was based on the appearance of dark orange staining, which corresponded to the deposition of DAB deposition, as compared with the negative and positive controls, revealing the presence of a monoclonal antibody recognizing kainic acid-type glutamate receptors (GluR 5, 6, 7). More detailed information can also be found in the work of Pulido [20].

### 2.6. DA levels in brain and liver

To measure DA levels in the brain and liver, nine individuals were exposed to PBS or 9.0 mg DA kg^−1^ bw. After 1, 2, and 4 hours of exposure, the brain and liver of three of the individuals were removed as described above, weighed, and then lyophilized.

DA was extracted from the reconstituted tissues of *S. aurata* [21] and proteins were extracted with perchloric acid using a previously described method [22]. Recovery experiments were carried out to evaluate the efficiency of the extraction method. For this purpose, brain and liver samples were spiked with 50 ng of DA. The mean recovery values were 80% for brain samples and 90% for liver, which indicated the presence of a matrix effect that affected these recovery values. The amount of DA in the tissues was determined by HPLC-UV [21] as summarized in Table 2. DA concentrations were quantified on the basis of standard curves that used external DA standards, after correction for the amount of recovery.

## 3. Results

### 3.1. DA neurotoxicity

The mortality of *S. aurata* (n = 18) after i.p. injection with 9.0 mg DA kg^−1^ bw was 66.7 ± 16.7% (mean ± SD). Under these conditions, 50% of mortality occurred at 7.1 hours after exposure. No mortality was observed in the controls or after exposure to the other concentrations of DA.

No signs of toxicity were observed in the controls. In some individuals exposed to 0.45 mg DA kg^−1^ bw, some disturbances in behavior, characterized by vertical swimming, *i.e.*, ascending and descending in the water column, were observed. The remaining individuals continued to show gregarious behavior, with the fish remaining in the centre of the water column or near the bottom of the aquarium. Similarly, in some individuals exposed to 0.9 mg DA kg^−1^ bw, disturbances in behavior were observed. However, for this treatment, the disturbances corresponded to neurotoxic signs, such as swimming in a circle, in a spiral, or upside down. The observed disturbances in behavior were reversed after 4–5 hours of exposure for the 0.45 mg DA kg^−1^ bw dose and after 12 hours for the 0.9 mg DA kg^−1^ bw dose. In contrast, neurotoxic signs were observed in all individuals exposed to 9.0 mg DA kg^−1^ bw from 20–30 minutes after i.p. injection. These signs, swimming in a circle, in a spiral, or upside down, were reversed after 24 hours of toxin exposure in the surviving individuals. The alterations in swimming behavior occurred in the following sequence. At first, the fish remained near the bottom of the aquarium, to one side, but were incapable of staying in the expected position. Subsequently, the individuals became distributed uniformly in the aquarium and showed severe circle swimming behavior, during which the fish were strongly impelled by the caudal fin. Finally, the fish remained near the surface of the aquarium, upside down, floating or slightly impelled by the pectoral fins. Moreover, an increase in the reactivity of *S. aurata* to various stimuli, visual and auditory, was observed. Surviving animals were monitored over a four week period and all showed signs of complete recovery.

### 3.2. Light microscopy and immunohistochemistry

Light microscopy studies revealed no major differences between the analyzed organs of the controls and those of the individuals exposed to DA.

**Figure 1:**
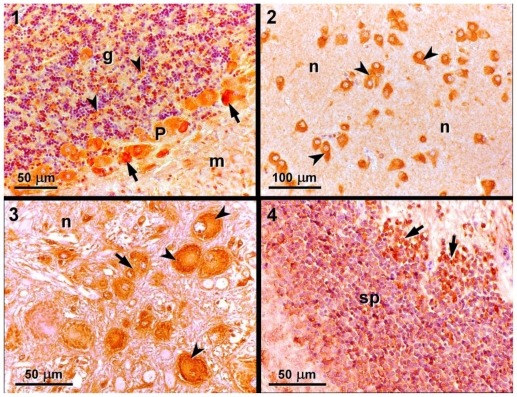
Brain sections of *S. aurata* showing positive immunoreactivity to the monoclonal antibody MAB379, which recognizes KA-type glutamate receptors (GluR5, 6, and 7): (**1**) Cerebellar cortex comprising the granular layer (g), Purkinje cell layer (P) and molecular layer (m). The cerebellar glomeruli (arrowheads) and Purkinje cell bodies (arrows) showed strong immunoreactivity; (**2**) Diencephalon comprising neuropil (n) and immunoreactive neuron cell bodies (arrowheads); (**3**) Myelencephalon comprising neuropil (n), glial cells (arrows), and neurons with peripherial Nissl bodies showing strong immunoreactivity (arrowheads); (**4**) Mesencephalon showing a layer with small immunoreactive neurons (arrows) in the stratum periventricularis (sp).

**Figure 2:**
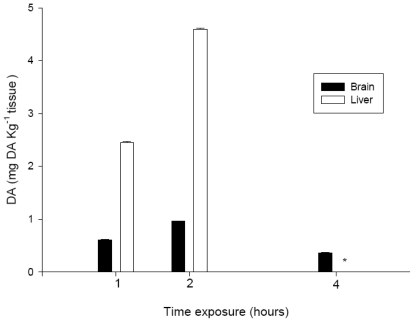
Domoic acid (DA) content (mg DA kg^−1^) in brain and liver extracts from *S. aurata* after i.p. injection of 9 mg DA kg^−1^ bw. * No evaluation possible due to coelution; Values of DA content represent the average ± SD from two replicate treatments.

The monoclonal antibody MAB379, was used for immunohistochemistry of brain sections obtained from fish injected with PBS alone. Immunopositivity for MAB379 was observed with different intensities (Figure 1). The negative controls showed no immunostaining. The Purkinje cell bodies, which are present in the cerebellar cortex, showed strong immunoreactivity, whereas the cerebellar glomeruli, which are located in the granular layer, showed moderate immunoreactivity (Figure 1-1). Several immunoreactive neuron cell bodies were identified in the diencephalon (Figure 1-2). The neuropil, which is a dense intricate felt work of interwoven fine glial processes, fibrils, synaptic terminals, axons, and dendrites that is interspersed among the bodies of the nerve cells and of the glial cells, was found to be immunonegative. Moreover, in the myelencephalon (Figure 1-3), strong immunoreactivity was observed in neurons that contained peripheral Nissl bodies, which are known to correspond to ribosomes in large aggregates and rough endoplasmic reticulum. Furthermore, in mesencephalon, a layer in the stratum periventricularis that corresponded to small neurons was found to show a moderate positive staining (Figure 1-4).

### 3.3. DA levels in the brain and liver

The mean (±SD) concentrations of DA that were detected by HPLC-UV in extracts of the brain of *S. aurata* after exposure by i.p. injection to 9.0 mg DA kg^−1^ bw were 0.61 ± 0.01, 0.96 ± 0.00, and 0.36 ± 0.01 mg DA kg^−1^ tissue at 1, 2, and 4 hours, respectively (Figure 2).

The mean (±SD) concentrations of DA in extracts of liver from *S. aurata* after 1 and 2 hours of exposure were 2.45 ± 0.02 and 4.59 ± 0.03 mg DA kg^−1^ tissue, respectively (Figure 2). The DA content in liver extracts after 4 hours of exposure was not evaluated, due to the presence of an unknown compound after extraction that had the same retention time and polarity as DA. This compound was not found in any of the fish that were exposed to PBS alone.

## 4. Discussion

In this study, DA-induced neurotoxicity in fish was assessed with respect to mortality, the occurrence of neurotoxic signs, and by observation using light microscopy. Furthermore, the amount of DA that was present in liver and brain extracts from *S. aurata* was quantified.

The structural similarity of DA to glutamic acid and, in particular, KA, suggests that DA can activate the KA and AMPA subtypes of glutamate receptor [23]. However, the mechanism of neuronal stimulation in response to DA has not been well clarified. Studies in primary cortical cultures have shown that DA acts via high affinity binding to AMPA- and KA-sensitive glutamate receptors to produce excitotoxic cell death [24]. The initial cause of excitotoxicity is an increased concentration of extracellular glutamate, which results in over-activation of local ionotropic glutamate receptors. This leads to an influx of Ca^2+^, which results in a failure of the cell to maintain intracellular ion homeostasis, and this in turn triggers cell death [25]. In addition, cytosolic Ca^2+^ levels have been reported to increase in hippocampus pyramidal neurons after exposure to DA [26]. DA has also been shown to induce excitation in cultured rat hippocampal neurons [27].

In this study, we did not find any major morphological changes that were induced by a single dose of DA. In contrast, Tryphonas and Iverson [28] reported that the i.p. administration of a single dose of 4 mg DA kg^−1^ to rats resulted in acute damage. Typical vacuolation of neuropili, hydropic cytoplasmic swelling of resident astrocytes and nerve cell hyperchromasia and shrinkage occurred. DA-induced neuronal cell loss (>80%) as well as the loss of neuronal cell bodies and degeneration of dendrites, in mice have been reported [24]. On the other hand, vacuolation of the neutropil and hypercromasia in the hippocampus, hypothalamus, area prostema, and the inner layers of the retina were noted post-mortem in primates that had been given DA at 0.5 mg DA kg^−1^intravenously or 4 mg DA kg^−1^ i.p. [29].

The fact that we did not observe permanent tissue damage in *S. aurata* by light microscopy, and that the toxic signs observed after exposure to DA were reversible, suggest that neuronal recovery occurred in the affected individuals. In our study, typical neurotoxic signs were observed in all individuals exposed to 9.0 mg DA kg^−1^ after 20–30 minutes. The variety of toxic signs observed in *S. aurata* that were exposed to a single dose of 9.0 mg DA kg^−1^, for example swimming in a circle, in a spiral, or upside down, were consistent with those reported previously for other fish genera after being injected with DA i.p. [15,17]. The systemic administration of DA to rats at a single dose of 1.3 mg kg^−1^ also caused a variety of toxic signs, which included stereotypic hind limb behaviors, scratching, and death [30]. Similarly to our study, these authors also reported a significant exaggerated auditory startle in rats exposed to DA.

Likewise, a study on the behavioral responses of mice after the administration of mussel extract i.p., observed a variety of responses, which included hypo activity, sedation, rigidity, scratching, head weaving, loss of postural control, tremors, convulsions, and death [23]. Similar neurotoxic effects to those observed in rodents have also been reported in non-human primates, but are dominated by gagging and vomiting. Cynomolgus monkeys that were exposed to a single dose of 4 mg DA kg^−1^ showed severe vomiting, hypothermia, acute pulmonary edema, and death within four hours [29].

The time course of observation of the neurotoxic signs was relevant for the interpretation of other data collected, such as the concentration of DA in the tissue extracts. At two hours after i.p. administration, the concentration of DA in the brain and liver extracts had increased, which indicated the bioavailability of DA in the systemic circulation. Conversely, at four hours, the amount of toxin detected in the brain had decreased again, to a value that was lower than that detected at one hour after exposure. These data are in agreement with the observed time frame for the recovery of normal behavior for all DA treatments, *i.e.*, after four hours of exposure, and also with findings published in the literature. Lefebvre *et al.* [17] indicated that DA is present in fish for only a short time period. The amount of DA that we detected in the liver after one or two hours of exposure indicated that the toxin was absorbed well from the coelomic cavity into the blood through capillaries, as had been expected due to the high solubility of DA in the water. The degree of toxicity exhibited, and the amount of DA detected in the tissues that were analyzed, also indicated that most of the toxin entered the systemic circulation directly. Moreover, the concentration of DA in extracts from the brain, showed that a portion of the DA that was administered i.p., migrate to the brain causing the above mentioned neurotoxic signs.

Very limited data is available in the literature regarding the absorption, distribution, metabolism, and excretion of DA in non-mammal models. Truelove *et al.* (1996) reported that in rats to which 5 mg DA kg^−1^ bw was administered by oral gavage (o.g.), only 2% of the dose was excreted in the urine [31,32].

Data from neurotoxicity assays revealed that the susceptibility of *S. aurata* to i.p. exposure to DA was similar to that of other fish, such as anchovies, coho salmon, and Atlantic salmon. The suggested lower susceptibility to DA toxic effects shown in the individuals that survived at 9.0 mg DA kg^−1^ treatment, might be in accordance with the absence of neurotoxic effects in leopard sharks, that have been exposed to concentrations of DA that were three times higher than those used in this study [14]. An overview of different factors that might modify cell injury associated to DA excitotoxicity, can be found in literature [20]. Although the surviving individuals in our study appeared to recover completely with no apparent lesions, more detailed studies are required in order to assess the effects of DA on brain metabolic activity or ultrastructural features.

In this study, we detected positive immunoreactivity to the monoclonal antibody MAB379, which recognizes the KA-type glutamate receptors (GluR5, 6, 7) in the brain of *S. aurata*, specifically in the cerebellar cortex, diencephalon, myelencephalon, and mesencephalon. This indicates that these regions of the brain contain at least one of the KA-type glutamate receptors, which are a target for DA. Immunohistochemistry studies performed in leopard sharks identified similar patterns of immunoreactivity in the same regions of the brain [14].

In nature, the exposure of *S. aurata* to sublethal concentrations of DA via the trophic food chain might compromise behaviors such as the capture of prey, predator avoidance, courtship, and mating. Nevertheless, the studies have shown that DA toxicity is much higher by i.p. injection than by oral intake on other fish species [17].

Taken together, the immunohistochemical studies and the behavioral observations enable a putative association between the two sets of findings to be established. The cerebellum, which showed strong immunoreactivity for the MAB379 antibody, is known to be involved in the maintenance of positional equilibrium, which was clearly affected in the *S. aurata* exposed to DA. In particular, the fish metencephalon is involved in processing information that is important for maintaining the positional equilibrium of the organism and for the refinement of motor action. These functions were clearly affected in individuals exposed to 9.0 mg DA kg^−1^ bw. Moreover, the myelencephalon operates primarily at the reflex level, given that it contains the centre for visceral, auditory, and proprioreceptive reflexes [33]. The myelencephalon is also the area associated with sound reception and integrates the taste and auditory senses [34]. These functions were affected in *S. aurata* that were exposed to 9.0 mg DA kg^−1^ bw, as revealed by the increased reactivity of individuals to visual and auditory stimuli.

In conclusion, the data obtained in this study indicate that a single, sublethal dose of DA does not seem to result in any acute damage to the brain tissue of the gilthead seabream *S. aurata*. Similarly, the behavioral changes that occurred after the injection i.p. of a single, sublethal dose of DA were found to be reversed fully after 24 hours.

## Figures and Tables

**Table 1:** Experimental setup used to evaluate the effects of domoic acid (DA) in the gilthead seabream *Sparus aurata*, three replicates were used in each treatment.

Experimental setup		Time (hours)	Number of fish per aquarium
Examination of DA neurotoxicity	Behavioral observations	0.5 and 2	6
	Mortality	24	6
Light microscopy and immunohistochemistry		24	2
DA analyses		1, 2, and 4	3

**Table 2:** HPLC-UV conditions for the analysis of domoic acid.

**HPLC Instrument**	PV-980 Jasco
**Column**	Reversed Phase Phenomenex luna C18, 5 μm, 100A ODS3, 250 × 4.6 mm
**Mobile Phase**	Acetonitrile:Water 12% (v/v) with formic acid 0.2% (v/v)
**Flow rate**	1 mL min^−1^
**UV detector**	Jasco UV-1575
**Wavelength**	242 nm
**Injection Volume**	20 μL
**Data Analysis**	Borwin software
